# Strain comparisons in inhibitory discrimination learning and novel object recognition procedures

**DOI:** 10.1016/j.physbeh.2021.113557

**Published:** 2021-10-15

**Authors:** L. Waite, C. Bonardi, C.W. Stevenson, H.J. Cassaday

**Affiliations:** a School of Psychology, University of Nottingham,UK; b School of Biosciences, University of Nottingham,UK

## Abstract

Strain differences in visual abilities and exploratory tendencies can confound rats’ performance in cognitive tests of learning and memory. In the present study we compared the performance of albino Wistar and pigmented Lister Hooded rats in appetitive conditioning and recognition memory procedures, specifically within-subjects inhibitory learning (*A*+ /AX-) and novel object recognition (NOR) variants. The inhibition task included an excitatory training stage and summation and retardation tests. Difference scores were used to help control for individual variation in baseline nosepoke responding. NOR was tested after a 10 min delay, following 24hr delay and using a recency variant. Discrimination ratios were used to control for individual variation in exploratory activity. In the inhibitory learning procedure, Lister Hooded showed more magazine activity prior to stimulus presentations than Wistar rats but this was a transient effect restricted to day 1 of excitatory training. There was no strain difference in associative learning at the excitatory training stage. The Wistars went on to show some performance advantage at the inhibitory discrimination stage and marginally stronger retardation test performance. In the NOR tasks, there was no significant effect of strain on cognitive performance, but the Wistars showed some advantage in the 10 min delay variant, whereas in the 24hr delay and relative recency NOR variants, the Lister Hooded rats showed some advantage. Overall the results of the present study confirm the suitability of Wistar rats for use in associative learning and basic NOR procedures.

## Highlights

• Behavioural comparisons of albino Wistar and pigmented Lister Hooded rats.
• Within-subjects inhibitory learning (*A* +/AX-) and novel object recognition variants.
• Wistars showed clearer inhibitory discrimination than Lister Hooded rats.
• Novel object recognition discriminations showed strain differences in consistency.
• Wistars are good for associative learning and basic object recognition procedures.

## 1 Introduction

Rodents are widely used in translational studies of behaviour and neuroscience. Rat studies have typically preceded mouse studies and results from mouse and rat studies have not always been found to be similar. Moreover, within species there can be strain differences in emotionality and/or cognition which influence task performance. There are a number of widely used strains of laboratory rat which have typically been inbred to reduce variability. However, the standardisation of individual strains has had the additional effect of widening the differences between strains [2]. Breeding line and even the barrier from which the rats are sourced have also been found to influence performance in behavioural tasks [17], but strain differences have been more widely reported. In particular differences between albino and pigmented rats are to be expected because of their differences in visual acuity and there may also be non-specific strain differences in motivation and/or exploratory activity.

### 1.1 Wistar and Lister Hooded strains

The albino Wistar strain [6] is widely used in psychopharmacology and behavioural neuroscience studies. Indeed the gold standard atlas for the rat brain was based on Wistar anatomy [28, 29]. Whereas Wistar rats are a popular choice for neuroscience and physiology research in general, for behavioural studies, pigmented Lister Hooded rats are often used as an alternative, both because Lister Hooded rats are viewed as easier to train due to their typically higher activity levels [7] and because of their higher visual acuity, which is important for tasks with a visual element [19]. In common with other albino rodents, Wistar rats have particularly poor eye sight [32, 33]. Once a strain has been selected for use in a particular task there can be a reluctance to switch strains, in case strain differences confound direct comparison of novel results with those previously published. Thus selection of strain depends on a balance of considerations. Moreover, whilst relatively low levels of activity and/or visual acuity may be limiting they do not necessarily preclude good behavioural performance.

In the present study, we compared performance of Lister Hooded and Wistar rats in (1) an inhibitory associative learning procedure [34, 39, 40] and (2) two novel object recognition (NOR) task variants [11], [12], [13].

### 1.2 Inhibitory associative learning

In Pavlovian inhibitory learning procedures, the expectation of an outcome is inhibited by the presence of a qualifying stimulus. Building on the basic classical conditioning procedure in which a conditioned stimulus (CS) signals an outcome (unconditioned stimulus, US), a further stimulus (the conditioned inhibitor) signals the omission of the expected US. For example if, after a number of pairings of a CS (*A*+) with a US, A is paired with another stimulus X and the US is omitted (AX-), subjects learn that X signals the absence of the US, i.e. it becomes a conditioned inhibitor [27]. Inhibitory learning has been successfully demonstrated in a large number of studies [34, 39, 40] including in human participants [16, 18, 21]. Nevertheless, and despite its great promise for translational studies, inhibitory learning has been a neglected area of research [36, 37]. The present study adapted a within-subjects procedure, developed for use in rodents, to measure both inhibitory and excitatory learning for each animal [5], using a design closely based on that used in an earlier human study [18]. Such within-subjects associative learning procedures are important because they promote the reduction of animal usage. Since the variety of auditory cues available in automated conditioning boxes is limited (animals generalise between them and they cannot be played simultaneously) the use of visual cues is necessary.

### 1.3 Novel object recognition

NOR tasks rely on rats’ spontaneous preference for investigating a new object over one that is already familiar. The preference for the novel object (shown by increased exploration of the new relative to the familiar object) suggests that recognition provides some basis for the discrimination. On the other hand, if the animal explores both objects equally or shows a preference for the familiar object over the novel one then it is inferred that recognition memory is impaired [35]. NOR variants are widely used in psychopharmacology and behavioural neuroscience studies because they require no training or food restriction to motivate responding and are both high-throughput and reliable [8, 12, 13].

NOR variants have been widely exploited in order to investigate different aspects of memory, including its formation, acquisition, consolidation and retrieval, and have provided an invaluable tool to investigate the neural substrates of the mechanisms of memory [3, 13]. Moreover, using different sets of objects, animals can be tested repeatedly in the different variants. Both levels of activity and visual acuity might be expected to influence the levels of exploration shown in NOR tasks. However, as discrimination is shown by the relative preference for a novel over a familiar object, any strain differences arising from general differences in activity are taken into account. Objects are selected to be easily discriminable based on gross features and previous studies conducted in the same laboratory have shown that Wistar rats can perform well in NOR tasks [23, 24, 30, 31]. In the present study, the same animals tested in the inhibitory learning procedures went on to complete three NOR task variants: the standard NOR variant tested at two retention delays and the recency variant [10, 11].

The 24hr delay variant tests the effect of increased retention demand on NOR performance and performance under increased memory load has been used to distinguish the neural substrates of consolidation/retrieval as distinct from the object encoding which is also required to perform at short retention delays [23, 30, 31]. The recency variant tests discriminations based on the relative familiarity of individual objects, and relies on medial prefrontal cortex (PFC), which is not a key substrate for basic NOR [4, 15, 22]. To our knowledge whilst there have been previous studies of strain differences in NOR [14, 38], strain differences in performance of the recency variant have yet to be examined.

### 1.4 Hypotheses to be tested

The systematic comparison of the performance of Lister Hooded and Wistar rats in the inhibitory learning and NOR tasks was done with the objective to test the evidence for the presumed superiority of the Lister Hooded strain in such tasks. Lister Hooded rats are generally reported to be more active; increased exploratory tendencies would be consistent with the potential to show superior cognitive performance, and unquestionably their visual acuity will be higher. However, if experimental stimuli and other task parameters are appropriately selected, levels of visual acuity and activity may not be limiting.

## 2 Methods

### 2.1 Animals

Experimentally naïve adult male rats, Wistar and Lister Hooded (Charles River, UK Ltd) were age matched (49–55 days) at the time of purchase (*n* = 16/strain). Upon arrival, their weight ranges were 231–280 g (mean 257 g) for the Lister Hooded and 264–337 g (mean 299 g) for the Wistar rats.

Rats were housed in groups of four, in double decker individually ventilated cages (IVCs) (462 mm x 403 mm x 404 mm; Techniplast) on a 12:12hr light/dark cycle (lights off at 19.00 hrs) at a temperature of 21 ± 1 °C. Prior to behavioural experiments, food (Teklad Global 18% Protein Rodent Diet, Envigo) and water were provided ad libitum. After arrival, rats were handled daily for 7 days whilst they acclimatised to the laboratory. Animals were placed on food restriction, whilst maintaining 80% free feeding weight, 2 or 3 days (for the Lister Hooded and Wistar groups respectively) prior to the start of Experiment 1 (taking into account the differences in the natural growth curves of each strain according to Charles River Strain Growth Charts). The mean experiment start weight (when food restriction commenced) was 319 g for the Wistar (284–353 g) and 281 g for the Lister Hooded rats (255–305 g).

In Experiment 1, once each squad had completed the experimental session they were returned to the home cage and given their daily food ration (minimum 5 g rodent diet per 100 g body weight; calculated as the total weight of all 4 animals in a cage/100 × 5). At the end of Experiment 1, all the rats were returned to ad libitum feeding. No food restriction was required for Experiment 2. All procedures were approved by the University of Nottingham Local Ethical Review Committee and conducted in accordance with the UK Animal Scientific Procedures Act 1986, Project Licence number: PPL P4C629C86.

### 2.2 Experiment 1: inhibitory associative learning

#### 2.2.1 Design

After a pre-exposure phase in which animals were exposed to all the stimuli to eliminate any unconditioned suppression, they were trained that each of two auditory stimuli, A and C, predicted sucrose delivery. Additional trials were then introduced in which A was accompanied by a visual stimulus, X; no sucrose was delivered on these AX trials. In this way X signalled the omission of the sucrose otherwise signalled by A, and so should acquire inhibitory properties, and the ability to counteract the effects of stimuli predicting sucrose. Thus the first indicator of inhibitory learning would be lower rates of responding during AX than A in this inhibitory training phase. Two further tests were then conducted to rule out alternative explanations of these results (Rescorla, 1980). In the summation test the ability of X to suppress the level of conditioned responding elicited by a different excitatory stimulus, C was evaluated. This was achieved by comparing levels of responding to C in compound with either the putative inhibitor X or a novel visual control stimulus, Y (i.e. CX versus CY). If X had acquired inhibitory properties then CX should elicit lower levels of conditioned responding than CY. This was followed by a retardation test, in which the rate of acquisition of excitatory conditioning to putative inhibitor X and control stimulus Y was compared. If X was an inhibitor it should acquire associative strength more slowly than Y. The experimental design is shown in Table 1.

**Table 1:** Experimental design and stimulus presentations.

Day 1	Days 2–7	Days 8–11	Day 12	Days 13–17
Pre-exposure	Excitatory training	Inhibitory discrimination	Summation test	Retardation test
6 A- 6 C-	15*A*+	10*A*+	5 A-	5 A-
6 X- 6 Y-	15 *C*+	5 *C*+	15 CX-	15 *X*+
		20 AX-	15 CY-	15 *Y*+

Experimental stages and schedule of stimulus presentations for the inhibitory learning task. For each stage the number and stimulus types are given with ‘-’ denoting non-reinforced trials and ‘+’ denoting reinforced presentations which were followed by 2 sucrose pellets. Stimuli A and C were the white noise and click respectively. Stimuli X and Y were either a constant right light or flashing left light (counter-balanced).

The within-subjects experimental design necessitates the use of a variety of experimental stimuli including some use of visual stimuli. However, these visual stimuli include temporal (flashing versus constant) as well as positional cues (the right light versus the left light) which should make them sufficiently salient to detect, even for albino rats. Importantly, the design also allows us to test for any differences in excitatory learning which could confound examination of inhibitory learning, because to demonstrate inhibitory learning requires an earlier stage of excitatory conditioning (*A*+) to set up the expectation of the outcome which is subsequently omitted in the presence of the inhibitor (AX-). The effects of non-specific effects on activity are reduced by the use of difference scores, to adjust for differences in responding seen prior to the CS presentations which could relate to strain differences in activity.

#### 2.2.2 Apparatus

Eight identical modular test chambers (MED Associates ENV-008) were used for all conditioning and testing procedures. Each steel chamber measured 20 × 24 × 30 cm, and had a Plexiglas rear wall and Plexiglas door, with the door being secured by a latch on the front. The conditioning chambers were housed in a ventilated noise-attenuating shell that measures 74 × 38 × 60 cm (MED Associates CT-ENV-016MX). Each chamber was equipped with a food magazine located on the right hand side wall of the chamber into which pellets could be delivered by a pellet dispenser (Model ENV-203). Head entry into the food magazine for the retrieval of a pellet was recorded by an infrared photobeam break, and each beam break was recorded as a response. The reinforcer comprised delivery of two sucrose pellets.

A 2.8-W house light, the bottom half of which was shielded, was located 11 cm above the food cup, and was switched on at all times for all experiments. There were two 2.8 W jewel lights, one 2.5 cm from each side of the food magazine. The right light was illuminated throughout its scheduled presentations, whereas the left one was always pulsed (0.33 s on and 0.33 s off). A speaker, to enable the delivery of auditory stimuli, was located on the top right of the back wall, opposite the food magazine. The auditory stimuli were a white noise and a 10 Hz click, both at 74 dB (scale A, measured near the food magazine). A was the noise and C the click. For half the rats in each strain, X was the constant light and Y the flashing light, and for the remainder the reverse. The floor was a shock grid (not in use) with 20 steel bars, 1 cm apart and 1 cm above a sawdust tray.

#### 2.2.3 Procedure

Rats were individually assigned to a conditioning box for the duration of the experiment and completed the below experimental stages. Throughout rats were run in squads of 6, and received a single training session each day. There was also 1 day of home cage exposure to the sucrose pellets reward to reduce neophobia: 5 g sucrose pellets replaced 5 g of the rodent diet (following pre-exposure, see below). Sucrose pellets were placed in each cage in a glass dish at the same time as the daily ration of rodent diet. All stimuli were presented for 20 s and preceded by a 20 s preCS period during which responding was also recorded. The inter-trial interval (ITI) was 40 s plus a 40 s variable interval component (from an exponential distribution) taking the ITI up to an average of 80 s. The different trial types in each of the experimental phases were presented in a semi-random order.

Pre-exposure: This was carried out one day prior to excitatory training so rats were habituated to their experimental boxes and to reduce unconditioned suppression to the experimental stimuli to be used in subsequent stages of the experiment. There were six presentations of each of A, C, X and Y. No sucrose rewards were delivered during pre-exposure.

Excitatory training: During each of the six sessions of this stage rats received 15 presentations of A and 15 presentations of C, each of which was followed by sucrose reward.

Inhibitory discrimination training: Inhibitory training was conducted over 4 days. Rats continued to receive pairings of A and C with sucrose, but in addition experienced unreinforced trials in which A was presented in a simultaneous compound with X. There were 10 A trials, 5 C trials and 20 AX trials in each of these sessions.

Summation test: The single summation test session comprised 15 presentations of each of CX and CY, and also five of A (to maintain continuity with the previous training stages); no reinforcers were delivered during this session.

Retardation test: In each of the five retardation test sessions there were 15 trials with X and 15 of Y, both of which were followed by sucrose reward. In addition, to ensure that not all stimulus presentations were followed by food, 5 nonreinforced A trials were also presented.

### 2.3 Experiment 2: novel object recognition

#### 2.3.1 Design

All three NOR variants had the same three basic stages: habituation, sample exposure and test, with an additional sample exposure for the recency variant described below. In the habituation session, the rats were free to explore their allocated arena for a period of 10 min. In the sample exposure, conducted on the following day, rats were again placed in their allocated arena and allowed to freely explore two identical objects for 5 min. At test, rats were subsequently exposed to one familiar object (identical to those encountered at the preceding sample stage) and one object which was either novel, or identical to objects presented at the earlier sample stage in the recency variant. The test stages were conducted after a specified delay (10 min, 15 min or 24hr) following completion of the sample exposure(s). NOR was shown as greater exploration of the novel (or least recently presented) over the familiar object. The three task variants were run sequentially with fixed selections of objects in the order described below.

#### 2.3.2 Apparatus

Four identical rectangular arenas made from opaque plastic and measuring 30 × 40 cm with a height of 54 cm provided the environment in which the objects were presented, with a transparent Perspex sheet placed on top of the arenas to ensure that no animals escaped. An overhead camera was used to film the rats’ exploratory behaviour for later scoring and analysis. The object stimuli were made from materials unlikely to retain any odour deposits (glass, metal and plastic) and each individual object had three duplicates to further eliminate the role of odour traces. The objects selected (bottles, containers and flasks of different sizes and shapes) had previously been found to support good NOR performance in the same arena [23, 24, 30, 31]. With the exception of their duplicates, the objects all differed in shape, size and colour. The designation of the object identities selected for the novel versus familiar conditions was counterbalanced.

Objects were attached (with Blu Tack) to the base of the arena, to prevent them being displaced or tipped over by the animals, equidistant from the sides and from each other. Exploration of an object was defined as pointing the nose at an object at a distance no greater than 1 cm and actively exploring the object by sniffing or otherwise interacting with the object.

#### 2.3.3 Procedure

Each rat was assigned to a specific arena for the duration of the experiments and the members of each squad (of 4 cage mates) were simultaneously placed into the centre of their individual arenas. Upon completion of the experimental sessions, all animals were returned to their home cages with their respective cage mates and returned to their keeping room (or an adjoining holding area between sample and test phases). The behavioural arenas and objects were cleaned with ethanol between squads, to remove any scent odours. The rats were all tested sequentially and with the same object selections, with intervals of 1–4 days between each procedural variant and in the order described below.

NOR after a10 min  delay: At a single sampling stage, rats were exposed to one of two pairs of identical novel objects, with counterbalancing so that the designation of an object as familiar was not confounded with its intrinsic properties. Once in the arenas, animals were left to freely sample the objects for 5 min before being returned to their home cage. Ten minutes later the rats were re-introduced to the experimental arena, but now at the test stage they were exposed to one of the objects familiar from sampling versus an exemplar of the objects to which they had no previous exposure. The position of the novel object and the identities of the objects selected as novel versus familiar were counterbalanced within tasks, but the pairs of objects in use were fixed for each task variant. Rats were again left to freely explore the objects for a period of 5 min after which they were returned to their home cage. Exploration was scored for the full 5 min of the sample exposure and the first 3 min of the test exposure [23, 24, 30, 31].

NOR after a 24 hour delay: Procedures were identical to those described above, using different sets of objects, again counterbalanced so that the designation of an object as familiar was not confounded with its intrinsic properties. However in this variant there were 24 hrs between the sample exposure and the test stage.

Recency after a15 min  delay: Procedures were identical to those above but there were two sample stages (separated by 1 hr) and test followed 15 min after the second sample exposure. At test, the choice was between objects sampled more or less recently, with counterbalanced allocations to the sample exposures so that relative recency was not confounded with any other properties of the objects in use.

### 2.4 Data analysis

Data were analysed by Analysis of Variance (ANOVA) in mixed factorial designs using SPSS software version 15.0, with the between-subjects factor of strain (Wistar or Lister Hooded). Planned comparisons were used to determine the presence or absence of behavioural effects for each of the strains. In both experiments, individual differences in baseline activity were taken into account (see below).

#### 2.4.1 Experiment 1

In the inhibitory conditioning experiment the measure of responding during each stimulus or stimulus compound was a *difference score*, computed as the average rate of responding (in responses per minute; rpm) during each particular trial type in each session, after subtraction of the rpm for the corresponding preCS periods. This CS-preCS score represented the extent to which stimulus presentation elevated responding over baseline levels. Rates of preCS responding, averaged over all trial types in each session, were also compared to help ensure that the difference scores were not contaminated by systematic strain differences in preCS responding.

The repeated measures factors were: days to assess the course of acquisition in the excitatory training, inhibitory discrimination training and retardation test stages of the inhibitory learning procedure, and stimulus to assess the rats’ responding during the different trial types. Stimulus was the only repeated measures factor for the one-day summation test. All analyses of the difference scores were focused on the key discriminations of interest: *A*+ vs AX- at inhibitory acquisition; CX- vs CY- at summation; *X*+ vs *Y*+ at retardation [1]. Stimuli presented in filler trials designed to maintain responding were not included in the analyses (see Table 1). Significant two-way interactions were explored with simple main effects analysis, using the pooled error term for between–subject comparisons. Follow up analyses to examine acquisition in each of the strains separately were used to assess whether the discriminations were individually significant for each strain.

#### 2.4.2 Experiment 2

In the case of NOR, ANOVAs of the raw exploration scores used familiarity as a factor so time spent at the novel object was considered in relation to the time spent at the familiar object. The repeated measures factors were familiarity (at 2 levels) to assess choice exploration of the novel (or less recently experienced) object and minutes (at 3 levels) to assess how levels of exploration changed over the first 3 min of the test sessions. NOR was demonstrated as significantly greater exploration of the novel over the familiar object following ANOVA of the raw scores. Independent samples t-tests were used to test for any strain differences in exploration at the sample stages of the NOR variants.

Further analyses used discrimination ratios which were computed as the time spent exploring the novel object divided by time spent exploring both objects during the choice phase, and provided an alternative method to take differences in baseline exploration into account and to allow statistical comparison with the ratio value which reflects no preference for the novel object (0.5). Discrimination ratios were calculated separately for each of the first three minutes of exploration at test. A ratio of 0.5 (reflecting no preference) was substituted for missing values arising from minutes in which there was 0 s exploration of the objects (neither was explored): two such substitutions were made for min 3 of the recency variant; one such substitution was made for min 3 of the 24hr variant. Analysis of the min-by-min discrimination ratios (which adjust individually and min-by-min for differences in exploration of the familiar object) also used minutes (at 3 levels) as a repeated measures factor to assess how levels of exploration changed over the first 3 min of the test sessions. One sample t-tests were used to check whether the NOR discrimination ratios were significantly different from the 0.5 value which reflects indiscriminate exploration of the two test objects. NOR was demonstrated by discrimination ratios significantly above 0.5.

Sample exploration data was lost for four animals (recency variant; one of the samples only). To get the best estimate of any strain difference at the sample stage the exploration times were averaged across minutes, objects and samples (for the recency variant), using just the available second sample data for the four rats missing sample 1 exploration times.

## 3 Results

### 3.1 Experiment 1: inhibitory learning

#### 3.1.1 Excitatory training

Nosepoking preCS: At the start of training the Lister Hooded responded more than the Wistar rats, but this difference dissipated over the course of this stage. ANOVA with Days and Strain as factors revealed effects of both Strain, *F*(1,30) = 4.58, *MSe* = 16.11, *p* = 0.041, and Days, *F*(5,150) = 13.86, *MSe* = 5.44, *p* < 0.001, and an interaction between these two factors, *F*(5,150) = 3.33, *MSe* = 5.44, *p* = 0.007; exploration of the interaction revealed that the groups differed on day 1, *p* = 0.017, but not on any of the remaining days, smallest *p* = 0.11 for day 2. There were no differences in the preCS by the stimulus type to follow, all Fs < 1. This transient strain difference in baseline responding is thus unlikely to compromise our interpretation of the difference scores in this stage. The mean rates of preCS responding are shown in Table 2, from which it is evident that rates of preCS responding declined over sessions, as the rats learned that the CS presentations – rather than background cues – were the best predictors of food delivery.

**Table 2:** preCS responding.

Day	EXC1	EXC2	EXC3	EXC4	EXC5	EXC6
**Lister**	9.41 (0.91)	8.39 (0.88)	5.47 (0.66)	4.03 (0.48)	5.63 (0.73)	4.09 (0.74)
**Wistar**	5.81 (0.60)	6.01 (0.57)	4.08 (0.55)	4.12 (0.54)	5.40 (0.74)	4.15 (0.51)
						
	**INH1**	**INH2**	**INH3**	**INH4**		
**Lister**	3.10 (0.47)	3.45 (0.51)	4.91 (0.84)	4.49 (0.90)		
**Wistar**	3.10 (0.40)	2.71 (0.32)	3.62 (0.49)	2.95 (0.35)		
						
	**SUM**					
**Lister**	1.94 (0.44)					
**Wistar**	1.03 (0.17)					
						
	**RET1**	**RET2**	**RET3**	**RET4**	**RET5**	
**Lister**	9.25 (0.44)	8.73 (1.48)	7.65 (1.62)	6.32 (1.25)	5.91 (1.39)	
**Wistar**	8.33 (1.01)	10.40 (1.80)	12.79 (2.00)	9.83 (1.44)	9.06 (1.67)	

Group mean rates for each session in the excitatory training (EXC), inhibitory training (INH), summation test (SUM) and retardation (RET) test sessions. The standard errors of the means are shown in brackets. There was a transient significant difference in preCS responding on day 1 (grey shaded).

**Fig. 1:**
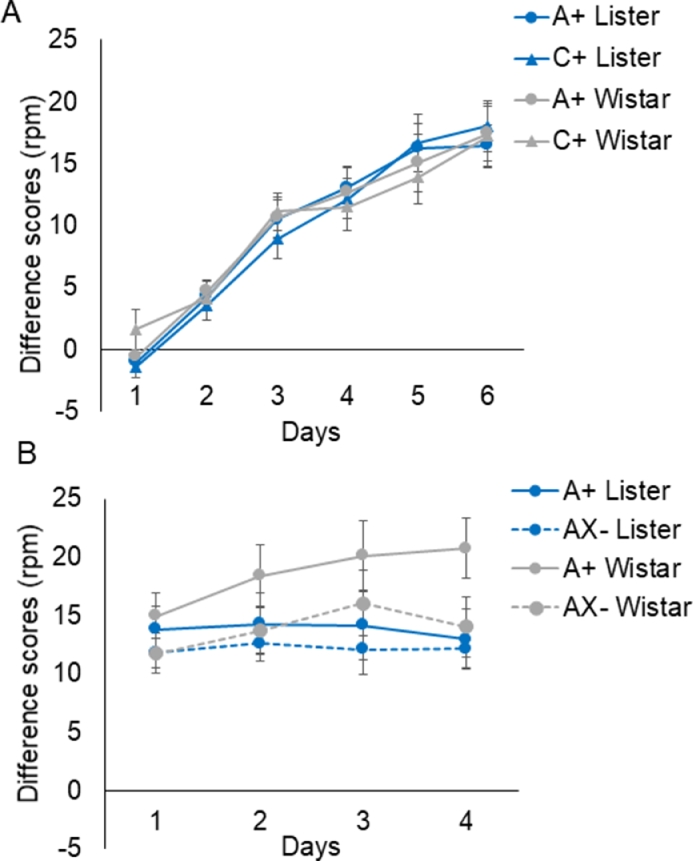
Group mean difference scores (CS-preCS responding) computed as the average rate of responding (in responses per minute; rpm) during each particular trial type in each session. Panel A shows the 6 day excitatory training phase (*A*+, *C*+) and panel B shows the 4 day inhibitory discrimination phase (*A*+, AX-). Error bars show the standard errors of the mean.

CS-preCS: Group mean difference scores for the six sessions of excitatory training are shown in Fig. 1A. It is clear that rats of both strains learned to respond to the excitors A and C, and this increase was very similar in the two groups. ANOVA of the difference scores showed a main effect of Days, *F*(5,150) = 72.55, *MSe* = 40.57, *p* < 0.001, reflecting increased responding over the course of the session. Critically there was no effect or interaction involving Strain, largest *F*(5,150) = 1.31, *MSe* = 9.84, *p* = 0.262, for the three-way interaction. Nothing else was significant, *Fs* < 1.

#### 3.1.2 Inhibitory discrimination training

Nosepoking preCS: Group mean preCS scores for the inhibitory training sessions are shown in Table 2; rates were again slightly higher in the Lister rats, but this difference was not significant, and ANOVA with Days and Strain as factors revealed only a main effect of Days *F* (3,90) = 4.58, *MSe* = 2.25, *p* = 0.005; nothing else was significant, largest *F*(1,30) = 1.78, *MSe* = 14.28, *p* = 0.19 for the effect of strain.

CS-preCS: Group mean difference scores for the inhibitory training phase are shown in Fig. 1B; the difference scores to A were consistently higher than those to AX in all sessions, and this difference was numerically more robust in the Wistar rats. ANOVA with Strain, Stimulus (A, AX) and Days as factors revealed a main effect of Stimulus, and a Stimulus x Strain interaction, *F*(1, 30) = 50.88, *MSe* = 12.54, *p* < 0.001, and *F*(1,30) = 11.85, *MSe* = 12.54, *p* = 0.002, respectively. The Stimulus x Strain interaction presumably arises because discrimination between A and AX was overall clearer in the Wistars (Fig. 1B). With respect to the acquisition of the discrimination, the Stimulus x Strain x Days interaction was significant in the linear trend, *F*(1,30) = 5.45, *MSe* = 35.25*, p* = 0.026, in the absence of significant residual variation [1]. This means that the slopes of the acquisition functions were different by stimulus and strain.

Follow up analyses to examine acquisition in each of the strains separately showed both a main effect of Stimulus, *F*(1,15) = 51.40, *MSe* = 701.02, *p* < 0.001, and a Days x Stimulus interaction, *F*(3,45) = 3.02, *MSe* = 7.83, *p* = 0.048, for the Wistars, and a main effect of Stimulus, *F*(1,15) = 7.47, *MSe* = 85.40, *p* = 0.015, but no Days x Stimulus interaction, *F* < 1 for the Lister Hooded rats. As shown in Fig. 1B, both strains showed discrimination but, counter to expectation, this effect developed more clearly over the days in the Wistar rats.

#### 3.1.3 Summation test

Nosepoking preCS: The mean rates of preCS responding during this session were 1.94 and 1.03 rpm for Lister Hooded and Wistar rats respectively, and these did not differ, *F*(1,30) = 3.69, *MSe* = 1.78, *p* = 0.06.

**Fig. 2:**
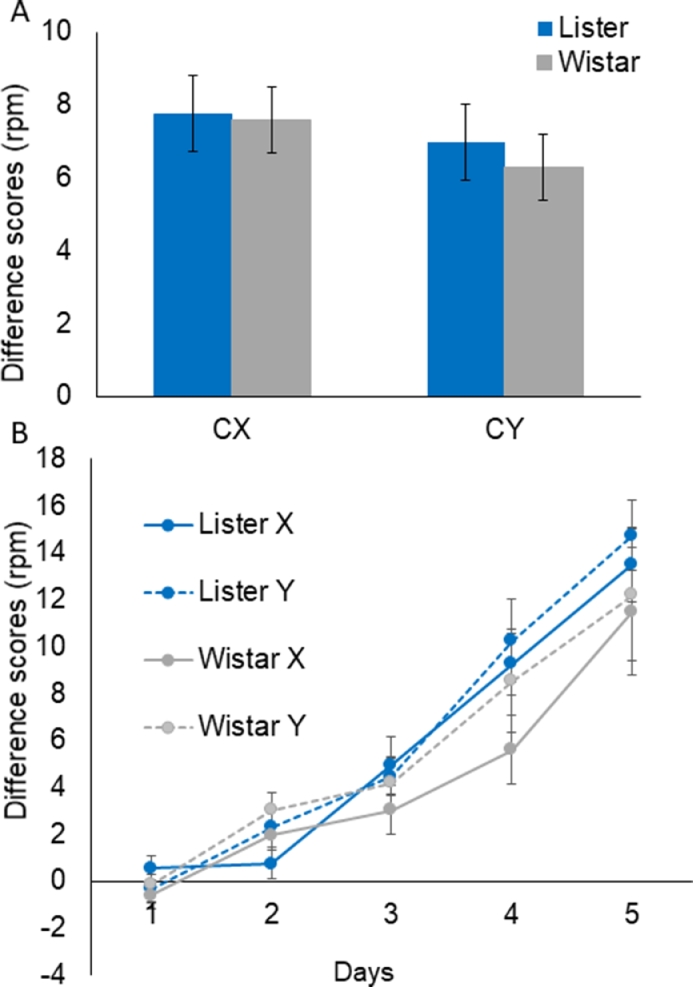
Group mean difference scores (CS-preCS responding) computed as the average rate of responding (in responses per minute; rpm). Panel A shows the one day summation test for CX and CY presentations and panel B shows the 5 day retardation test for X and Y presentations. Error bars show the standard errors of the mean.

CS-preCS: The results of the summation test are shown in Fig. 2A; there was in fact little sign of a difference in responding on CX and CY trials, or between the strains. ANOVA with Stimulus and Strain as factors revealed nothing significant, largest *F*(1,30) = 1.70, *MSe* = 10.52, *p* = 0.20 for the effect of Stimulus. Thus, according to this strict criterion, there was no evidence for conditioned inhibition by the summation test measure.

#### 3.1.4 Retardation test

Nosepoking preCS: The group mean preCS rates during these sessions are shown in Table 2; ANOVA revealed no effect of Strain, *F*(1,30) = 1.77, *MSe* = 142.50, *p* = 0.19, but a significant effect of Days, and an interaction between these two factors, *F*(4120) = 3.25, *MSe* = 11.93, *p* = 0.014 and *F*(4120) = 3.48, *MSe* = 11.93, *p* = 0.01 respectively. However, exploration of the interaction showed that the groups did not differ on any session, all *ps* > 0.2, confirming that differences in preCS responding were not compromising interpretation of the difference score data.

CS-preCS: There was evidence of learning across the retardation test session, and as expected this was clearly retarded for X versus Y presentations (shown as relatively lower responding to X). The Stimulus effect was significant by ANOVA restricted to the critical X (*M* = 5.04, SEM = 0.62) and Y (*M* = 5.92, SEM = 0.72) stimuli, F(1,30) = 4.69, *MSe* = 12.98, *p* = 0.038. There was also an overall effect of Day, *F* (4, 120) = 47.74, *MSe* = 37.03, *p* < 0.001; nothing else was significant, largest *F* (4, 120) = 1.35, *MSe* = 8.49, *p* = 0.26 for the Stimulus x Day interaction.

Because of the a priori interest in learning differences at retardation, follow-up analyses examined acquisition to the key stimuli (the inhibitor X vs the novel stimulus Y) separately for each of the strains. For the Wistar rats, there was a main effect of Days, *F*(4,60) = 14.56, *MSe* = 48.58, *p* < 0.001. The effect of Stimulus was now marginal for X vs Y, *F*(1, 15) = 4.54, *MSe* = 14.22, *p* = 0.050, and there was no Days x Stimulus interaction, *F* < 1. For the Listers, there was a main effect of Days, *F*(4,60) = 42.98, *MSe* = 25.47, *p* < 0.001. The effect of Stimulus was non-significant, *F* < 1, and there was no Days x Stimulus interaction, *F*(4, 60) = 1.28, *MSe* = 11.74, *p* = 0.287. Thus the Wistars showed a marginal performance advantage (as shown in Fig. 2B).

### 3.2 Experiment 2: novel object recognition

#### 3.2.1 NOR after a 10 min delay

**Fig. 3:**
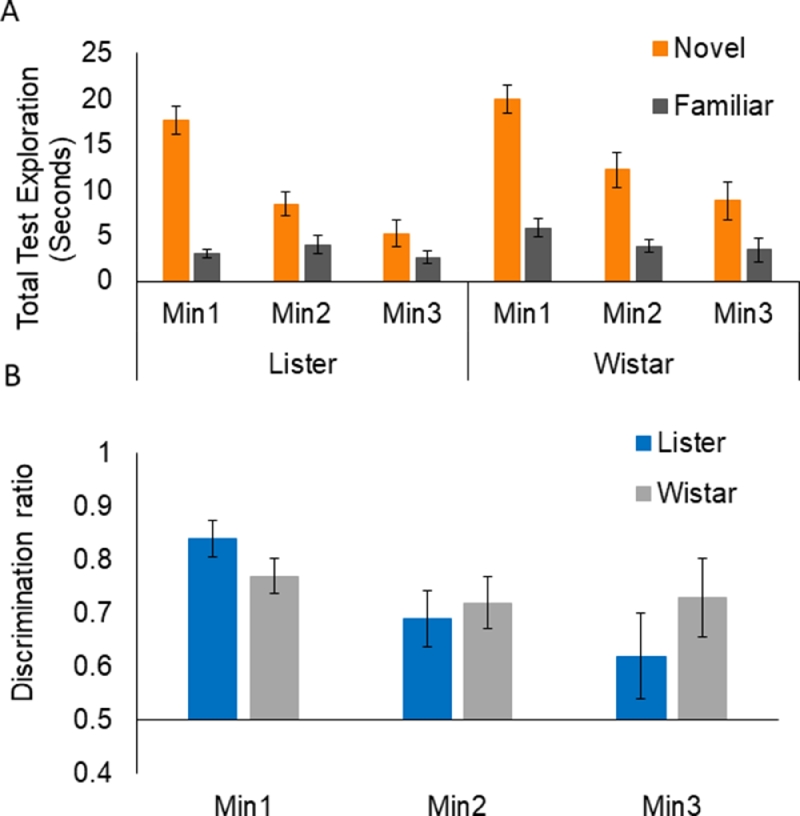
Object exploration over 3 min of test for the 10 min delay novel object recognition variant. Panel A shows group mean test exploration scores (in seconds) and panel B shows group mean discrimination ratio scores. Error bars show standard errors of the mean.

At the sample stage, the strains were well matched for overall exploration, *t*(30) = 0.73, *p* = 0.47. ANOVA of test exploration times with the familiarity factor showed a main effect of Min, *F*(2,60) = 31.41, *MSe* = 23.03, *p* < 0.001, because rats explored less in successive minutes of the test session (Fig. 3A). Importantly, there was an overall effect of Familiarity, *F*(1,30) = 73.92, *MSe* = 43.20, *p* < 0.001, and an interaction between Min and Familiarity, *F*(2,60) = 20.96, *MSe* = 23.12, *p* < 0.001. The main effect of Strain, *F*(1,30) = 7.02, *MSe* = 36.92, *p* = 0.013, arose because, at the test stage, the Listers *M* = 6.83/min explored overall less than the Wistars *M* = 9.16/min. However, there was no effect of strain on NOR performance as none of the interactions involving strain approached significance, *Fs* < 1. As can be seen in Fig. 3A, exploration of the novel object was higher than that of the familiar object for both strains across all 3 min and this difference reduced in successive minutes of the test session.

Analysis of the min-by-min discrimination ratios confirmed these conclusions. There was an effect of Min, *F*(2,60) = 3.37, *MSe* = 0.05, *p* = 0.041, in the absence of any effect of Strain, maximum *F*(2,60) = 1.49. As can be seen in Fig. 3B, both strains showed preferential exploration of the novel over the familiar object (shown as higher ratio scores) and this preference reduced over successive minutes of the test session. Planned comparisons by one-sample *t*-tests to determine whether performance was significantly above 0.5 (which reflects indiscriminate exploration of the novel versus familiar object) showed that this was the case in all but the 3rd min of test for the Listers: min 1, *t*(15) = 10.17, *p* < 0.001; min 2, *t*(15) = 3.67, *p* = 0.002; min 3, *t*(15) = 1.45, *p* = 0.17. The Wistars’ discrimination was sustained over the full test duration: min 1, *t*(15) = 8.47; *p* < 0.001; min 2, *t*(15) = 4.43, *p* < 0.001; min 3, *t*(15) = 3.16, *p* = 0.006. Thus despite the unexpected difference in the overall level of exploration at the test stage, both strains showed good NOR performance at the 10 min delay, with some marginal Wistar advantage in this the most basic variant of the task.

#### 3.2.2 NOR after a 24hr delay

At the sample stage of the 24hr variant, the strains showed some differences in exploration, *t*(30) = 2.656, *p* = 0.013, with the Wistars (*M* = 45.53; SEM = 3.25) on average exploring more than the Listers (*M* = 34.69; SEM = 2.48). This difference was not seen at sample exploration in the 10 min variant but the direction of the difference, with the Wistars exploring more, is the same as the difference in overall test exploration identified at the test stage in the 10 min variant. The ANOVAs to assess differential exploration of the novel versus familiar object at test take any test baseline differences into account (though, as discussed below, more exploration in the sample phase could in principle improve performance at test).

**Fig. 4:**
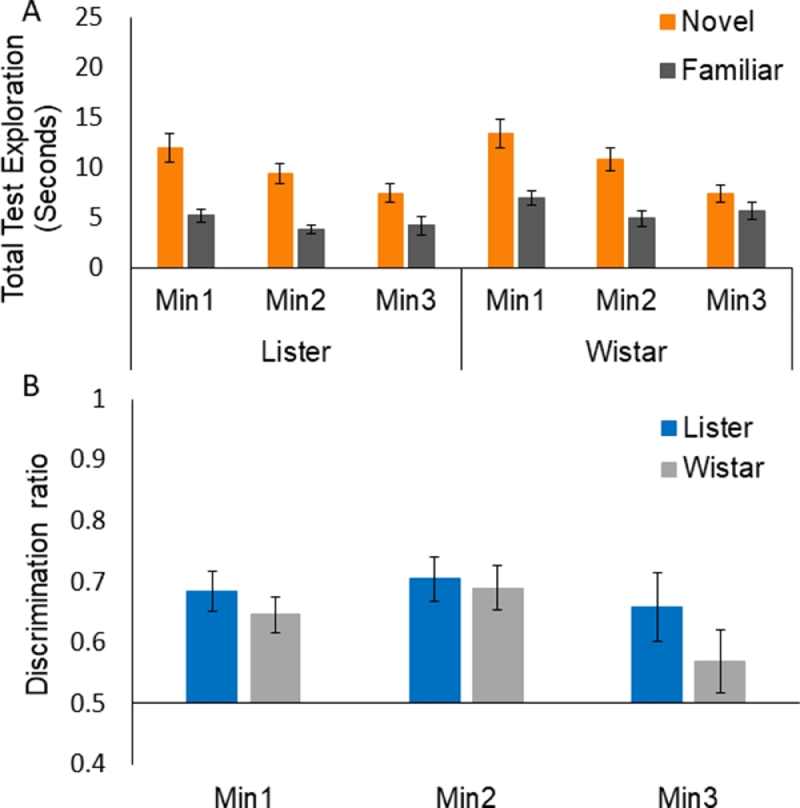
Object exploration over 3 min of test for the 24 hour delay novel object recognition variant. Panel A shows group mean test exploration scores and panel B shows group mean discrimination ratio scores. Error bars show the standard errors of the mean.

ANOVA of test exploration times with the familiarity factor showed a main effect of Min, *F*(2,60) = 16.99, *MSe* = 10.14, *p* < 0.001, because rats explored less in successive minutes of the test session. Importantly, there was an overall effect of Familiarity, *F*(1,30) = 65.70, *MSe* = 17.81, *p* < 0.001, and an interaction between Min and Familiarity, *F*(2,60) = 5.43, *MSe* = 13.77, *p* = 0.0007. There was no overall effect of strain and no interaction of strain on NOR performance as none of the interactions involving strain approached significance, *Fs* < 1. As can be seen in Fig. 4A, exploration of the novel object was higher than that of the familiar object in all 3 min of test and this difference was reduced by the 3rd min with increased exposure to the less familiar object.

Analysis of the min-by-min discrimination ratios showed no overall effect of Min, *F*(2,60) = 1.87, *MSe* = 0.30, *p* = 0.163, in the absence of any effects by Strain, Fs < 1. As can be seen in Fig. 4B, both strains showed preferential exploration of the novel over the familiar object (shown as higher ratio scores) and this preference tended to reduce by the 3rd min of the test session. Planned comparisons by one-sample t-tests to determine whether performance was significantly above 0.5 (which reflects indiscriminate exploration of the novel versus familiar object) showed that this was the case for each of the 3 min of test for the Listers: min 1, *t*(15) = 5.51, *p* < 0.001; min 2, *t*(15) = 5.72, *p* = 0.001; min 3, *t*(15) = 2.79, *p* = 0.014. Although not significantly different by ANOVA, the Wistars performance was less consistently above 0.5 at the 24hr delay: min 1, *t*(15) = 5.06, *p* < 0.001; min 2, *t*(15) = 5.15, *p* < 0.001; min 3, *t*(15) = 1.32, *p* = 0.208. Thus both strains showed NOR at the 24hr delay, but this effect was more robust in the Listers.

#### 3.2.3 Recency after 15 min delay

**Fig. 5:**
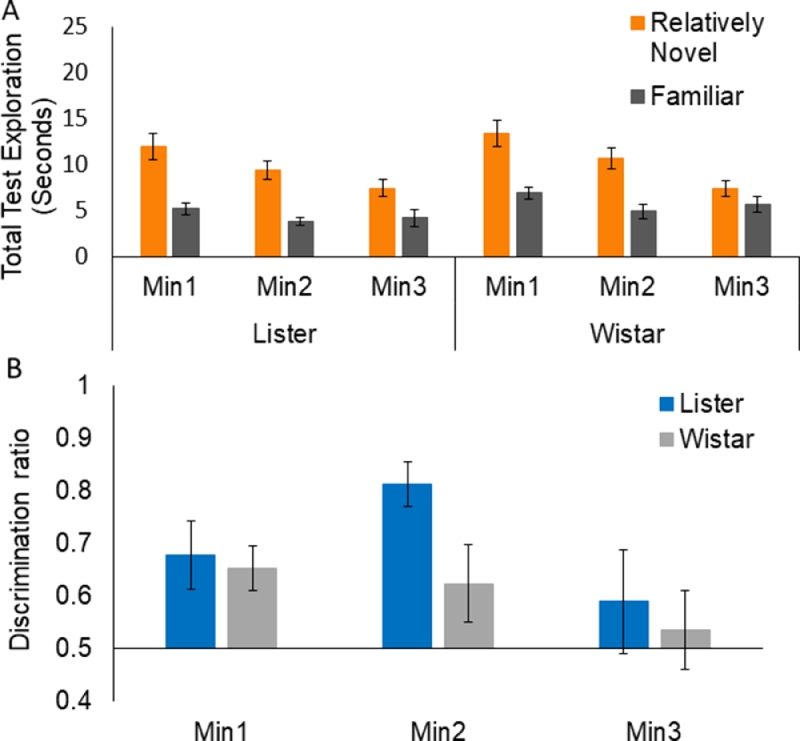
Object exploration over 3 min of test for the recency object recognition variant. Panel A shows group mean test exploration scores and panel B shows group mean discrimination ratio scores. Error bars show the standard errors of the mean.

There was no significant strain difference in total sample exploration in the recency variant, *t*(30) = 0.96, *p* = 0.345. ANOVA of test exploration times showed an overall effect of Familiarity, *F*(1,30) = 14.63, *MSe* = 84.09, *p* = 0.001 and an overall effect of Min *F*(2,60) = 3.62, *MSe* = 36.23, *p* = 0.033. No other effects or interactions were significant, maximum *F*(2,60) = 2.852, for Familiarity by Min. Thus there was no strain difference in NOR performance or in overall exploration at the test stage of the recency variant. As can be seen in Fig. 5A, exploration of the less recently exposed object was higher than that of the more recently exposed object. This effect tended to reduce by the 3rd min with increased exposure to the less familiar object (in the absence of a significant interaction, see above).

Analysis of the min-by-min discrimination ratios confirmed the conclusion that there was no strain difference in performance of the recency variant. As can be seen in Fig. 5B, both strains showed preferential exploration (shown as higher ratio scores) of the less recently explored (and hence relatively novel) object over the more recently seen (and hence relatively familiar) object, and this preference reduced by the 3rd minute of the test session. However, the ANOVA showed no significant effects, maximum *F*(2,60) = 1.06, for the effect of Min by Strain. Planned comparisons by one-sample t-tests to determine whether performance was significantly above 0.5 (which reflects indiscriminate exploration of the novel versus familiar object) showed that this was the case in all 3 min of the test for the Listers: min 1, *t*(15) = 2.80, *p* = 0.013; min 2, *t*(15) = 7.46, *p* = 0.001; min 3, *t*(15) = 2.64, *p* = 0.019. Performance was less robust in the Wistars as the discrimination ratios were significantly above 0.5 only in the first minute: min 1, *t*(15) = 3.65, *p* = 0.002; min 2, *t*(15) = 1.79, *p* = 0.094; min 3, *t*(15) = 2.02, *p* = 0.062. Thus although there was no significant effect of strain, the Lister Hooded rats showed some advantage in the relative recency variant.

### 3.3 Inter-rater-reliability

An independent experimenter rescored 50% of the sample and test stage object exploration for the 10 min delay variant. The re-scored results significantly correlated with the original scores (for the sample stage, *r* = 0.891, *p* < 0.001, for test stage exploration of the novel objects, *r* = 0.901, *p* < 0.001, and for test stage exploration of the familiar objects, *r* = 0.924, *p* < 0.001) indicating robust inter-rater reliability. A different independent experimenter scored 100% of the test exploration phase for the recency variant. The re-scored results significantly correlated with the original scores (for the test stage exploration of the familiar objects, *r* = 0.863, *p* < 0.001, and for the novel objects, *r* = 0.764, *p* < 0.001) again indicating robust inter-rater-reliability. No further checks of inter-rater-reliability were conducted as scoring was also done blind to object novelty. NOR scoring could not be conducted blind to strain because of the distinctive markings of the Lister Hooded rats.

## 4 Discussion

Both strains performed well, in both the inhibitory learning and novel object recognition tasks, but there were a number of differences in their profiles of performance. The Wistar rats showed some numerical performance advantage at the inhibitory discrimination stage (as illustrated in Fig. 1 and statistically supported by the significant interactions involving stimulus by strain). Importantly this difference in inhibitory discrimination learning could not be attributed to a prior difference in the excitatory learning which is fundamental to the expression of inhibitory learning. At the test stage, the overall effect of retardation was significant but when the strains were examined separately it was at the threshold for significance only in the Wistar, not the Lister Hooded rats.

There was no overall difference in performance by strain in any of the NOR variants, but the minute-by-minute discrimination ratios showed some strain differences in the consistency of the rats’ recognition memory over the three minutes of exploration which we routinely examine (because discrimination does not always persist as long as the 3rd minute). Analyses of the discrimination ratios tell us when performance is above chance and, by this criterion, the Wistars showed some advantage in the 10 min delay variant, whereas in the 24hr delay and relative recency NOR variants, the Lister Hooded rats showed some advantage (in that the discrimination ratios were more consistently above chance). Overall the results of the present study confirm that Wistar are at least as good as Lister Hooded rats for use in associative learning and basic NOR procedures.

In both experiments there was evidence to suggest that there were non-specific strain differences in motivation and/or exploratory activity. This was seen in Experiment 1 where the Lister Hooded nosepoked in the magazine more than the Wistar rats prior to the CS presentations and the food deliveries (on the preCS measure). However, the strain differences in exploratory responding seen in Experiment 2 were in the opposite direction. In Experiment 2, the Lister Hooded showed some *reduced* exploration compared to the Wistar rats, which is opposite also to the expected direction of effects based on both previous studies of home cage activity [7] and object exploration [14]. This direction of difference seen in the present study, seemingly suggesting reduced exploratory tendencies in the Lister Hooded rats, could relate to some compensatory strategy adopted by the Wistar rats, to explore up close objects that they were less well equipped to view at a distance outside the 1 cm range which defined exploration. Differences in the profile of this difference (seen at the test stage in the 10 min variant, the sample stage of the 24hr variant and not at all in the recency variant) could relate to the specific object selections which were fixed rather than counterbalanced across task variants. Although reasonably well-matched, it is to be expected that some objects would be more visually salient than others, particularly for rats with low visual acuity and the Wistar rats showed higher levels of object exploration in the NOR procedures.

Individual differences in activity (nosepoking or object exploration) were taken into account, by the use of difference measure scores in Experiment 1 and by the discrimination ratio scores in Experiment 2. The strain difference seen in Experiment 1 preCS was a transient and small effect, so differences in baseline levels of responding are not likely to have compromised the difference scores because of scaling effects. Moreover, the factorial ANOVAs of the results of the within-subjects designs, used to examine performance at the different stages of the inhibitory learning design and of the NOR variants, allow us to distinguish overall effects of strain from those which depend on the role of the stimuli presented or the novelty of the objects.

### 4.1 Inhibitory learning

There was no strain difference in associative learning at the excitatory training stage. Both strains went on to learn the inhibitory discrimination stage but, counter to expectation, this effect was more pronounced in the Wistar rats. These findings are in line with an earlier study which confirmed the suitability of the Wistar strain in a trace fear conditioning procedure which used a flashing light stimulus [25]. At the retardation test levels of conditioned responding to X were overall lower than to Y. This effect did not differ significantly by strain but follow-up analyses showed that the retardation test discrimination was marginally better in the Wistar strain. On the other hand, there was no evidence for inhibition in the summation test, with responding at similar levels to C regardless of whether it was presented with the putative inhibitor X, or the novel control stimulus Y, and this lack of discrimination was similar in the two groups. Thus the summation test was not passed using a stringent control for the effects of introduction of the CX- compound. To compare CX- versus CY- is a strong control, and as C- was not included at the summation test there was no conventional weak control in the present study.

It has previously been argued that for a stimulus to be a true conditioned inhibitor it is necessary to pass both the summation and retardation test. However, it has also been pointed out that many earlier studies of conditioned inhibition have not used full controls in both the retardation and summation tests, or may not have counterbalanced key stimuli correctly [26]. Moreover, whilst there have been studies of conditioned inhibition which address these limitations and both the summation and retardation tests are passed [9], these have been relatively few [26, 36, 37]. Thus the two test method remains an ideal which is not much put into practice. Recently it has been argued that studies of discrimination learning of the kind necessary to successfully demonstrate conditioned inhibition but which do not include (or do not pass) the formal tests to confirm conditioned inhibition are nonetheless informative in behavioural neuroscience, as has been the case in the safety signal literature [36, 37].

The appetitive task used in the present study has the huge advantage that it is within-subjects. Rather than comparing responding to the different stimuli between groups, rats act as their own controls in that the question is whether they differentiate between the stimuli within the learning sessions. These designs are very powerful, providing experimental control for any systematic differences in responding between strains (as well as individual variability) and with the further control of the difference scores to take preCS responses into account. However, the design presents quite complex discriminations and was conducted using stimuli from the same modality for X versus Y. Moreover, the one day summation session is a ‘one shot’ test with no scope for any further learning, so it is perhaps unsurprising that the rats failed to differentiate CX- and CY-. In contrast, the measures of retardation were repeated over 5 days and involved new learning.

### 4.2 Novel object recognition

Both strains performed well in the 10 min delay NOR variant. The Lister Hooded rats’ performance was significantly above chance in all but the 3rd min of test and the Wistars’ discrimination ratios remained above chance for the full test duration, reflecting sustained discrimination and suggesting some marginal Wistar advantage in this the most basic variant of the task. In the 24hr delay NOR variant, there was still no significant difference by strain. However, in this variant only the Lister Hooded rats’ discrimination ratios remained above chance for the full test duration, reflecting sustained discrimination and suggesting some marginal Lister advantage. The Wistars’ performance was above chance only for the first two minutes of test at the 24hr delay. In the most challenging relative recency variant, the Lister Hooded rats’ discrimination ratios remained above chance for the full 3 min scored at test. Whilst the factorial analyses again showed no significant differences by strain, the planned comparisons suggested that performance was less robust in the Wistar rats as their discrimination ratios were above chance only for the first minute of the test session of the recency variant.

These findings confirm earlier studies of strain differences in NOR [14, 38], and extend them to examine the recency variant which relies on different neural substrates. In terms of underlying mechanisms for the differential engagement of PFC, the recency variant, requiring the ability to discriminate objects, both familiar but experienced at different time points, may require some representation of the order in which the objects were encountered, i.e. memory for temporal order as such (e.g. [4, 15, 22]). However, if the rats preferentially explore the least recently seen object because the memory trace for that object is weaker than the most recently sampled object, they discriminate on the basis of relative recency rather than the order of occurrence of the objects [12]. It is difficult to differentiate between these two possible accounts of performance on the recency task [12, 24]. However, whatever the underlying psychological mechanisms, the recency variant presents additional cognitive challenges and relies on additional neural substrates which could in principle be the bases for strain differences.

In a slightly larger sample (*n* = 20; sham-operated) Wistar rats previously showed good discrimination in a recency variant [24]. However, in the latter study, the recency variant was conducted first so there was no possibility of proactive interference in episodic-like memory, following exposure to other similar objects in earlier conducted task variants [10, 11]. Taken together with our earlier findings, the present results confirm the suitability of Wistar rats for use in NOR procedures, with the reservation that naïve Wistars may perform better in the recency variant.

### 4.3 Limitations

The NOR object selections were not counterbalanced across the three task variants which were run sequentially rather than in a Latin square design (to reduce the risk of human error). However, the counterbalancing within each NOR variant is sufficient because the objective was to test for strain differences rather than to attempt to compare task difficulty as such. We have previously tested animals sequentially on different NOR variants [23, 24, 30, 31].

A full factorial study of the effects of strain, sex and age across a wider variety of cognitive tasks would allow for stronger conclusions but was beyond the scope of the present study. However, the within-subjects inhibitory learning procedure is new and the relatively strong performance of the Wistar rats suggests that they should perform similarly well in other within-subjects associative learning procedures. We furthermore examined the standard NOR variant at two retention intervals (10 min and 24hr) and we included the recency variant which was not included in previous studies of strain differences in NOR [14, 38]. These previous studies addressed sex differences, showing male advantage as measured by the discrimination index in both Lister Hooded and Wistar rats [14] and an effect of the oestrus cycle in females [38]. The effects of ageing are not so easy to address as the environmental factors confounded with ageing can make all the difference. In a longitudinal study of ageing male Wistar rats, we found that object exploration was overall reduced in middle aged rats and was further reduced when the rats were 6 months older. However, an initial NOR impairment (seen at a 24hr but not at a 10 min retention interval) showed recovery at the second point of testing [20]. These findings suggest that any strain differences by age seen cross-sectionally could similarly be overcome with enriched housing conditions and regular handling.

### 4.4 Conclusions and implications

Despite their undoubtedly poor visual acuity, male Wistar rats performed well in the Pavlovian inhibitory learning procedure and showed some performance advantage over the Lister Hooded strain. In the NOR variants, whilst it must be acknowledged that there were no significant differences by strain, the Lister Hooded rats' discrimination ratios suggested some performance advantage in the more challenging 24hr delay and relative recency NOR variants. There was no suggestion of any Wistar disadvantage in the 10 min delay NOR variant, on the contrary the Wistars showed a more sustained discrimination in the 10 min delay variant. Overall the results of the present study confirm the suitability of the Wistar strain for use in associative learning and basic NOR procedures. Visual acuity as such is less likely to be an issue in the case of relatively bright on-off and positional light cues, indeed being albino might rather increase sensitivity to such cues. Moreover, even in the case of the more visually demanding NOR tasks, the strain difference between variants could be consistent with a difference in visual memory in the more challenging 24hr and recency variants rather than acuity as such, because the Wistar rats performed well at the 10 min delay.
